# Dihydroartemisinin ameliorates cerebral I/R injury in rats via regulating VWF and autophagy-mediated SIRT1/FOXO1 pathway

**DOI:** 10.1515/med-2023-0698

**Published:** 2023-07-03

**Authors:** Qi Duan, Junxia Wu

**Affiliations:** Emergency Department, Nantong Rich Hospital, Nantong, Jiangsu, 226006, China; Emergency Department, The Sixth People’s Hospital of Nantong, No. 500 Yonghe Road, Gangzha District, Nantong, Jiangsu, 226000, China

**Keywords:** cerebral ischemia/reperfusion injury, brain microvascular endothelial cells, dihydroartemisinin, von Willebrand factor, SIRT1/FOXO1 pathway, autophagy

## Abstract

Dihydroartemisinin (DHA) has been found to inhibit the expression of von Willebrand factor (VWF), a marker of endothelial cell injury, but its mechanism in cerebral ischemia/reperfusion (I/R) injury remains obscure. In this study, I/R model was constructed through middle cerebral artery occlusion (MCAO) in rats, followed by DHA administration. The effect of DHA on rat cerebral I/R injury was investigated by 2,3,5-triphenyltetrazolium chloride staining, hematoxylin and eosin staining, TUNEL staining, and Western blot. Brain microvascular endothelial cells (BMVECs) isolated from newborn rats were exposed to oxygen–glucose deprivation/reoxygenation (OGD/R), and then treated with DHA. The results showed that MCAO treatment induced infarction, nerve cell apoptosis, and brain tissue impairment in rats, which was mitigated by DHA. OGD/R inhibited viability and accelerated apoptosis of BMVECs, which was alleviated by DHA. I/R procedures or OGD/R up-regulated expressions of VWF, ATG7, Beclin1, and LC3-II/LC3-I ratio, while down-regulating Occludin, Claudin-5, ZO-1, P62, SIRT1, and FOXO1 expressions *in vivo* and *in vitro*; however, these effects of I/R procedures or OGD/R were offset by DHA. VWF overexpression reversed the above effects of DHA on OGD/R-induced BMVECs. In summary, DHA ameliorates cerebral I/R injury in rats by reducing VWF level and activating autophagy-mediated SIRT1/FOXO1 signaling pathway.

## 1 Introduction

The annually rising incidence of cerebral ischemic stroke (CIS) is consequent on population aging worldwide [1]. As a common neurological disorder, CIS is characterized by high rates of recurrence, disability, and mortality [2]. Revascularization therapy is now widely recognized as an effective treatment for cerebral infarction caused by the occlusion of main arteries in China and abroad. However, extensive data showed that patients with CIS are predisposed to cerebral ischemia/reperfusion (I/R) injury after revascularization, including increased vascular permeability, disruption of the blood–brain barrier (BBB), and cerebral edema, posing a threat to the life of patients [3,4]. Brain microvascular endothelial cells (BMVECs), connected by tight junction proteins, are the main cells that contribute to the formation of BBB [5]. A study has demonstrated that I/R-induced BMVEC impairment is the initial stage of BBB disruption, leading to poor prognosis of patients with CIS [6]. Hence, studying how to maintain normal function of BMVECs is an essential part of protecting the BBB, thus alleviating cerebral I/R injury.

Von Willebrand factor (VWF), a glycoprotein synthesized and secreted by vascular endothelial cells and megakaryocytes, has been early identified to be associated with vascular hemophilia, and is perceived as a marker of endothelial cell damage [7]. In recent years, numerous studies have confirmed that elevated VWF expression is a risk factor for vascular diseases [8–10]. Besides, VWF was found to be highly expressed in mice with liver I/R injury, and to exacerbate the disease progression [11]. Presently, the role of VWF in cerebral I/R injury has not been fully elucidated.

The main pathological mechanism of ischemic cerebrovascular disease is irreversible damage and death of brain neurons [12]. It is generally believed that necrosis and apoptosis are the primary processes of cell death. Notably, autophagy has been identified to be critical in a range of pathophysiological changes following cerebral I/R [13–15]. Autophagy is a cellular self-stabilization program that is highly conserved in eukaryotic evolution. In this process, autophagic vesicles encapsulate damaged organelles and proteins, and deliver them to lysosomes for degradation which in turn provides substrates for cellular metabolism [16]. Sirtuin1 (SIRT1)/forkhead box O1 (FOXO1) signaling pathway-mediated autophagy has been proved to be implicated in a variety of organ damages, including cerebral I/R injury [17–19]. Therefore, probing into the mechanisms that activate the SIRT1/FOXO1 signaling pathway to inhibit autophagy has become a topical issue in cerebral I/R injury research.

Dihydroartemisinin (DHA) is a semi-synthetic derivative of the natural compound artemisinin, which exerts assorted pharmacological effects such as anti-malaria, anti-oxidation, anti-inflammation, and anti-apoptosis [20–22]. An existing study has identified that DHA is capable of regulating multiple biological properties of endothelial cells and suppressing VWF expression [23]. Also, DHA has been proven to protect against myocardial I/R injury [24], but its role in cerebral I/R injury needs further study.

Here, the current study investigated the role of DHA in rat cerebral I/R injury, and whether its potential mechanisms are related to VWF expression and SIRT1/FOXO1 signaling pathway through *in vivo* and *in vitro* experiments.

## 2 Methods

### 2.1 Animals and ethics statement

This study was carried out on newborn Sprague Dawley (SD) rats (10 days old) and adult male SD rats weighing 240–310 g (Charles River Laboratories, Beijing, China). The rats were housed in a specific pathogen-free environment and given a standard diet and drinking water *ad libitum*. Animal surgical procedures were approved by the Committee of Laboratory Animals of Nantong Rich Hospital (CIR20201006), and performed in accordance with the Guideline for the Care and Use of Laboratory Animals.

### 2.2 Cerebral I/R procedures and drug administration

All rats were randomly divided into three groups: Control group, I/R group, and I/R + DHA group. The cerebral I/R model was constructed using middle cerebral artery occlusion (MCAO), as previously reported [25]. Briefly, the rats in the I/R group and I/R + DHA group were anesthetized with pentobarbital sodium (45 mg/kg, P-010; Whitehouse Station, New Jersey, Merck, USA). Then, a midline incision was created to expose the right common carotid artery, internal carotid artery, and external carotid artery (ECA). After the ECA was separated, a nylon suture (0.26 mm in diameter) was inserted into the lumen of the ECA for 18–20 mm until blunted distal end met resistance. Two hours later, the incision was sutured when the suture was withdrawn. The rats in the Control group received identical surgical procedures aside from the occlusion. Subsequently, 15 min later, 0.5 mL dimethyl sulfoxide (DMSO, 10% v/v, D8418, Merck, USA) and DMSO-dissolved DHA (0.1 mg/kg, C_15_H_24_O_5_, D140839, Aladdin, China) were used to treat rats in I/R group and I/R + DHA group, respectively, by tail vein injection [24]. Following 14 days of reperfusion, all animals were euthanized by anesthesia and cervical dislocation for histological analysis of brain tissues.

### 2.3 Histological analysis

To evaluate the neuroprotective effect of DHA on I/R rat brain, 2,3,5-triphenyltetrazolium chloride (TTC) staining was applied to calculate cerebral infarct volumes, as previously described [26]. The frozen brain tissues were cut into sections (5 μm) and stained with TTC solution (17779, Merck, USA) at 37°C in the dark for 30 min. Then, the TTC-stained sections were fixed with 4% paraformaldehyde (abs9179, Absin, China). Infarction areas, which are manifested by a lack of red staining on the sections, were quantified using Image J software (vision 1.8.0, National Institutes of Health, Bethesda, MD, USA).

For morphological evaluation, hematoxylin and eosin (HE) staining was carried out using HE Staining Kit (G1121, Solarbio, China). According to the protocols, the frozen sections were stained with hematoxylin at room temperature for 10 min followed by differentiation. Afterward, the sections were subjected to bluing treatment, then were stained with eosin for 2 min and washed with water. A microscope (CX23, Olympus, Japan) was finally used for observation under ×100 magnification.

Terminal deoxynucleotidyl transferase-mediated dUTP-biotin nick end labeling (TUNEL) staining was conducted using a TUNEL kit (MK1015, Wuhan Boster Biological Technology, Ltd). A microscope was applied to observe five randomly selected fields in the injury site of brain tissues in a blinded manner and determine the TUNEL-positive neurons.

### 2.4 Cell culture

BMVECs were isolated from newborn SD rats as previously described [6]. In brief, the sacrificed rats were scrubbed with 75% ethanol (E111991, Aladdin, China) and the cranial cavity was exposed. Next, the cerebral cortex was separated after removal of the meninges and blood vessels. The cortex was minced, and collagenase and dispase two-step combined dissociation (10269638001, Roche, Basel, Switzerland) was used to digest the tissues. By centrifugation at 1,000×*g* for 5 min at 4℃, BMVECs were aspirated and suspended with Dulbecco’s Modified Eagle Medium/Nutrient Mixture F-12 (DMEM/F-12, 11320033, Thermo Fisher, Waltham, MA, USA) supplemented with 20% fetal bovine serum (FBS, 10091155, Thermo Fisher, USA) and basic fibroblast growth factor (70 ng/mL, TL-401, Wolcavi, China). Lastly, BMVECs were seeded (1 × 10^5^/cm^2^) in a gelatin-coated plastic dish at 37°C with 5% CO_2_ for 2 days of subculture.

### 2.5 Oxygen–glucose deprivation/reoxygenation (OGD/R) treatment

The cultured BMVECs were treated with glucose-free medium (E600010-0500, Sangon Biotech, China) with 2% FBS in an incubator (37°C, 5% CO_2_ and 95% N_2_) as per guidance. Six hours later, the cells were transferred to the standard medium and incubated in a normoxic condition (25% O_2_, 5% CO_2_, and 70% N_2_) for 4 h. BMVECs cultured in the normoxic condition were used as the control.

### 2.6 Cell transfection and DHA treatment

VWF-overexpressing plasmids (Bes-mR-053889) and control plasmids (NC) were purchased from BersinBio (Guangzhou, China). BMVECs were transfected with VWF-overexpressing plasmids or control plasmids by Lipofectamine 3000 (L3000008, Thermo Fisher, USA) according to the specification. After OGD/R treatment, the cells were incubated with 25 μM DHA for 24 h [23]. In the end, five groups were constructed: Control group, OGD/R group, OGD/R + DHA group, OGD/R + DHA + NC group, and OGD/R + DHA + VWF group. The cells from above groups were subsequently subjected to quantitative real-time reverse transcription polymerase chain reaction (qRT-PCR), cell function experiments, and Western blot.

### 2.7 RNA extraction and qRT-PCR

Total RNA from BMVECs with different treatments was isolated with Trizol (9108, Takara, Japan) according to the manufacturer’s instructions, and then was reverse-transcribed using cDNA Synthesis Kit (6130, Takara, Japan). After that, qRT-PCR was conducted on a Real-Time System (CFX96, Bio-Rad, Hercules, CA, USA) with Fast SYBR Green Master Mix (4385612, Thermo Fisher, USA). The gene-specific primer sequences were VWF (forward: 5′-ATGGCCCTTTCCTGACCTAC-3′; reverse: 5′-GGATTAGGGTTGGCCCTGAG-3′) and glyceraldehyde-3-phosphate dehydrogenase (GAPDH, forward: 5′-TAATGCCGCCCCTTACCATC-3′; reverse: 5′-GGTGCAGCGATGCTTTACTT-3′). The conditions of PCR reaction were as follows: predenaturation at 95°C for 20 s and 40 cycles of amplification at 95°C for 10 s, annealing at 60°C for 20 s and extension at 60°C for 30 s. The relative expression of VWF was normalized based on the 2^−ΔΔCt^ method [27], with GAPDH serving as the endogenous control.

### 2.8 Cell counting kit 8 (CCK-8) assay

After OGD/R, BMVECs transfected with VWF-overexpressing plasmids or not were adjusted to the density of 2 × 10^3^ cells/well and seeded in a 96-well plate (100 μL/well). Following 24 h of incubation, the cells were subjected to DHA treatment and incubation with 10 μL CCK-8 solution (M4839, Abmole Bioscience, Houston, TX, USA) for 1 h. Thereafter, the optical density value was detected by a microplate reader (Synergy HTX, BioTek, Winooski, VT, USA).

### 2.9 Flow cytometry

Annexin V-FITC/propidium iodide (PI) Apoptosis Detection Kit (CA1020, Solarbio, China) was employed to detect cell apoptosis. In short, BMVECs treated with DHA for 24 h were collected at a concentration of 1  ×  10^5^ cells/mL, washed with cold phosphate buffered solution (PBS, abs9459, Absin, China), suspended with Binding Buffer, and centrifuged. Then, 100 μL cell suspension was incubated with 5 μL Annexin V-FITC at room temperature for 10 min away from light, and 5 μL PI for 5 min. The cells were mixed with 500 μL PBS, and apoptotic cells were analyzed using a flow cytometer (CytoFlex LX, Beckman Coulter, Miami, FL, USA).

### 2.10 Western blot

Total protein from rat cerebral cortex and BMVECs was extracted with a lysis buffer (BC-R327, Elabscience, China), and protein concentration was determined using BCA Protein Assay Kit (ab102536; Abcam, Cambridge, UK) according to the protocols. Equal numbers of protein samples (30 μg) were separated by 10% SDS-PAGE (P0670, Beyotime, China). After electrophoresis, the protein samples were transferred onto polyvinylidene fluoride membranes (P2438, Merck, USA), which were then treated with Tris buffered saline with Tween 20 (TBST)-dissolved in 5% skim milk (E-BC-R337, Elabscience, China) at room temperature for 1 h. Then, the membranes were cultured with diluted primary antibodies overnight (4℃). The next day, the membranes were washed with TBST and probed with secondary antibodies at room temperature for 1 h. Immunoblots were visualized by ECL Western Blotting Substrate (PE0010, Solarbio, China), and immunoreactivity was analyzed using a Tanon 5200 Imaging System (Shanghai, China). GAPDH served as the loading control. All antibodies used were as follows: VWF (1/1,000, 309 kDa, ab174290; Abcam, UK), Occludin (1/1,000, 59 kDa, ab167161; Abcam, UK), Claudin-5 (1 mg/mL, 23 kDa, AF5216; Affinity Biosciences, China), ZO-1 (1/500, 195 kDa, ab190085; Abcam, UK), LC3-I/LC3-II (1/2,000, 14, 16 kDa, ab192890; Abcam, UK), P62 (1/10,000, 62 kDa, ab109012; Abcam, UK), GAPDH (1/10,000, 36 kDa, ab181602; Abcam, UK), SIRT1 (0.125 µg/mL, 81 kDa, ab110304; Abcam, UK), FOXO1 (1/2,000, 70 kDa, ab70382; Abcam, UK), transcription factor 5 (ATF5) (1/2,000, 31 kDa, ab184923; Abcam, UK), autophagy-related gene 7 (ATG7) (1/10,000, 77 kDa, ab133528; Abcam, UK), Beclin1 (2 µg/mL, 52 kDa, ab217179; Abcam, UK), goat anti-rabbit IgG H&L (HRP) (1/2,000, ab205718; Abcam, UK), and goat anti-mouse IgG H&L (HRP) (1/2,000, ab205719; Abcam, UK).

### 2.11 Statistical analysis

Data were analyzed by Graphpad Prism 8.0 (GraphPad Software Inc., San Diego, CA, USA) and expressed as mean ± standard deviation. Differences of CCK-8 assay results among multiple groups were compared using two-way analysis of variance (ANOVA). Differences of other experiment results were analyzed using one-way ANOVA. Values of *p* < 0.05 indicated significant difference.

## 3 Results

### 3.1 DHA attenuated cerebral I/R injury and apoptosis in rats

**Figure 1:**
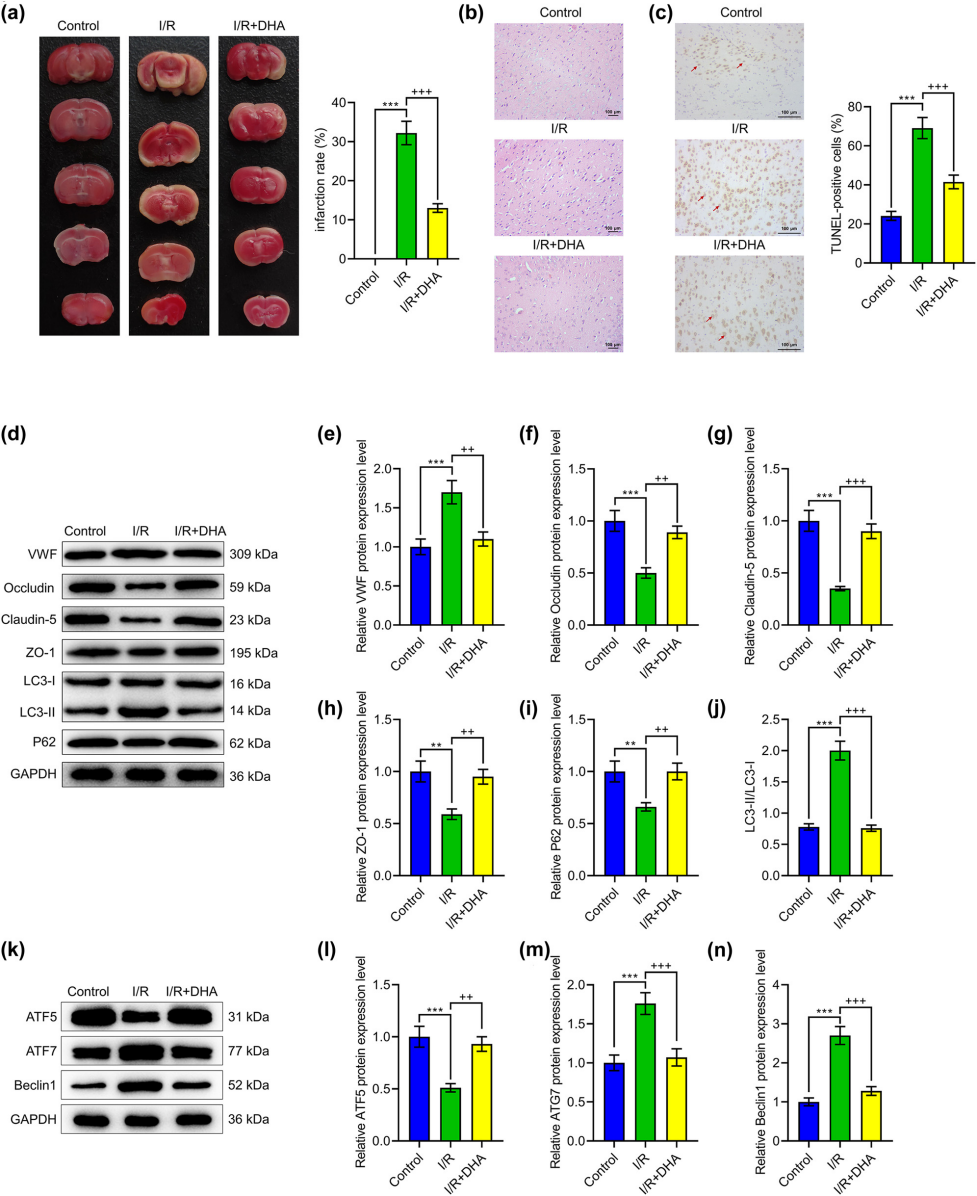
Effects of DHA on cerebral I/R injury *in vivo*. SD rats were subjected to MCAO to mimic I/R injury and then administrated with DHA (0.1 mg/kg), with unprocessed rats serving as the control. (a) Brain tissues were collected from sacrificed rats to calculate infarction area by TTC staining. (b) HE staining was applied to evaluate histopathological conditions in rat brain (magnification ×100, scale bar: 100 μm). (c) TUNEL staining indicated neuron nuclei (brown). Red arrows indicated TUNEL-positive neurons after I/R injury (magnification ×100, scale bar: 100 μm). (d–j) Western blot was employed to detect relative protein levels of VWF, Occludin, Claudin-5, ZO-1, LC3I, LC3II, and P62 in rat brain tissues. GAPDH was used as the loading control. (k–n) Western blot was applied to detect relative protein levels of ATF5, ATG7, and Beclin1 in rat brain tissues. GAPDH was used as the loading control. ^**^ *p* < 0.01, ^***^ *p* < 0.001 vs Control; ^++^ *p* < 0.01, ^+++^ *p* < 0.001 vs I/R, ischemia/reperfusion; DHA, dihydroartemisinin; VWF, von Willebrand factor; ATF5, transcription factor 5; ATG7, autophagy-related gene 7; ZO-1, zonula occludens 1; LC3, microtubule associated protein 1 light chain 3 alpha; GAPDH, glyceraldehyde-3-phosphate dehydrogenase; TTC, triphenyltetrazolium chloride; HE, hematoxylin and eosin.

As illustrated in Figure 1a, no infarction was observed in TTC-stained brain sections in the Control group. By contrast, cerebral I/R procedures induced a high infarction rate in the I/R group, but treatment with DHA in I/R-induced rats significantly reduced the infarction rate in IR + DHA group (Figure 1a, *p* < 0.001). The degree of rat brain injury after I/R was assessed by HE staining. The results revealed an intact tissue structure and normal neuron cells with clear membranes and nuclei in the Control group, but different degrees of interstitial edema, neuronal cell swelling, and nucleus disintegration in the I/R group. These conditions were mitigated by DHA administration in IR + DHA group (Figure 1b). In addition, TUNEL results indicated that DHA administration evidently reduced the number of TUNEL-positive cells in I/R-induced rats on day 14 following recovery (Figure 1c, *p* < 0.001). These results demonstrated that DHA administration exerted a protective effect by relieving the injury and apoptosis of nerve cells following IR.

### 3.2 DHA down-regulated VWF expression, protected BBB from breakdown, and inhibited autophagy in rat brain with I/R injury

Previous evidence indicated that VWF contributes to endothelial cell injury, which could be associated with autophagy-related SIRT1/FOXO1 pathway. Hence, we measured protein expressions of VWF, BBB-related proteins, and autophagy-related genes in rat brain tissues by Western blot. In the I/R group compared with the Control group, VWF, ATG7, and Beclin1 protein expressions and LC3-II/LC3-I rate were obviously elevated, yet Occludin, Claudun-5, ZO-1, P62, and ATF5 protein expressions were markedly lessened (Figure 1d–n, *p* < 0.01). In contrast, these trends were reversed by DHA treatment in the I/R + DHA group (Figure 1d–n, *p* < 0.01).

### 3.3 DHA promoted viability and suppressed apoptosis of BMVECs by down-regulating VWF expression

**Figure 2:**
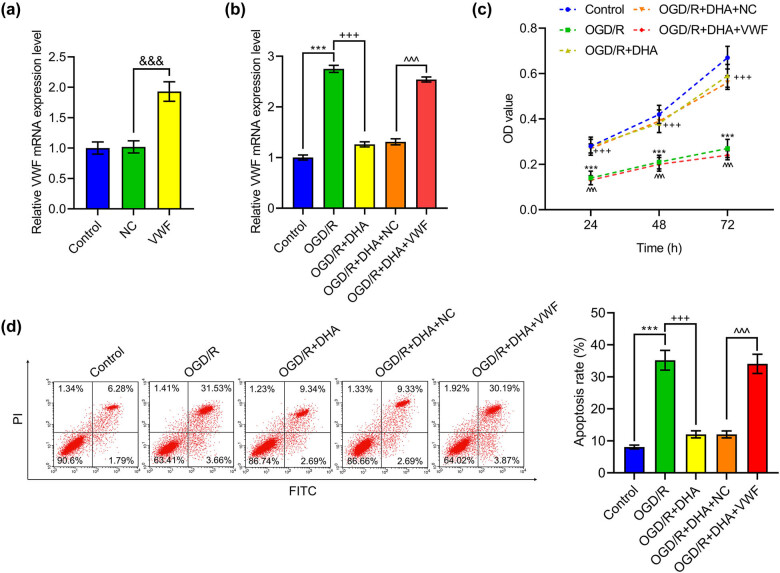
DHA promoted viability and repressed apoptosis of OGD/R-treated BMVECs by down-regulating VWF level. BMVECs isolated from newborn SD rats were transfected with VWF-overexpressing plasmids or control plasmids. OGD/R exposure was conducted on the cells with or without transfection, followed by 24 h 25 μM DHA treatment. (a and b) mRNA expression of VWF in BMVECs was determined by qRT-PCR. GAPDH served as the internal control. (c) Cell viability was assessed using CCK-8 assay. (d) Apoptotic cells were stained with Annexin V-FITC/PI and apoptosis rate was analyzed by flow cytometry. ^***^ *p* < 0.001 vs Control; ^+++^ *p* < 0.001 vs OGD/R; ^^^^^ *p* < 0.001 vs OGD/R + DHA + NC. BMVECs, brain microvascular endothelial cells; DHA, dihydroartemisinin; VWF, von Willebrand factor; OGD/R, oxygen–glucose deprivation/reoxygenation; qRT-PCR, quantitative real-time reverse transcription polymerase chain reaction; GAPDH, glyceraldehyde-3-phosphate dehydrogenase; CCK-8, cell counting kit 8; NC, negative control.

To figure out whether the effect of DHA on cerebral I/R injury is mediated by VWF, we carried out *in vitro* experiments using BMVECs exposed to OGD/R. The results of qRT-PCR unveiled that VWF plasmid remarkably elevated the mRNA level of VWF in BMVECs, indicating successful transfection (Figure 2a, *p* < 0.001). Furthermore, the results showed that OGD/R led to VWF overexpression, the effect of which was notably reversed after DHA treatment (Figure 2b, *p* < 0.001). In the OGD/R + DHA + VWF group compared with the OGD/R + DHA + NC group, the expression of VWF was significantly augmented (Figure 2b, *p* < 0.001). CCK-8 assay results unraveled that DHA treatment remarkably enhanced OGD/R-suppressed cell viability, which, however, was reversed by VWF overexpression (Figure 2c, *p* < 0.001). Moreover, the analysis of flow cytometry demonstrated that cell apoptosis was boosted by OGD/R, but was then inhibited after DHA treatment (Figure 2d, *p* < 0.001). However, VWF overexpression offset the protecting effect of DHA on cell apoptosis (Figure 2d, *p* < 0.001).

### 3.4 DHA inhibited SIRT1/FOXO1 pathway-mediated autophagy and improved tight junction of BMVECs by down-regulating VWF expression

**Figure 3:**
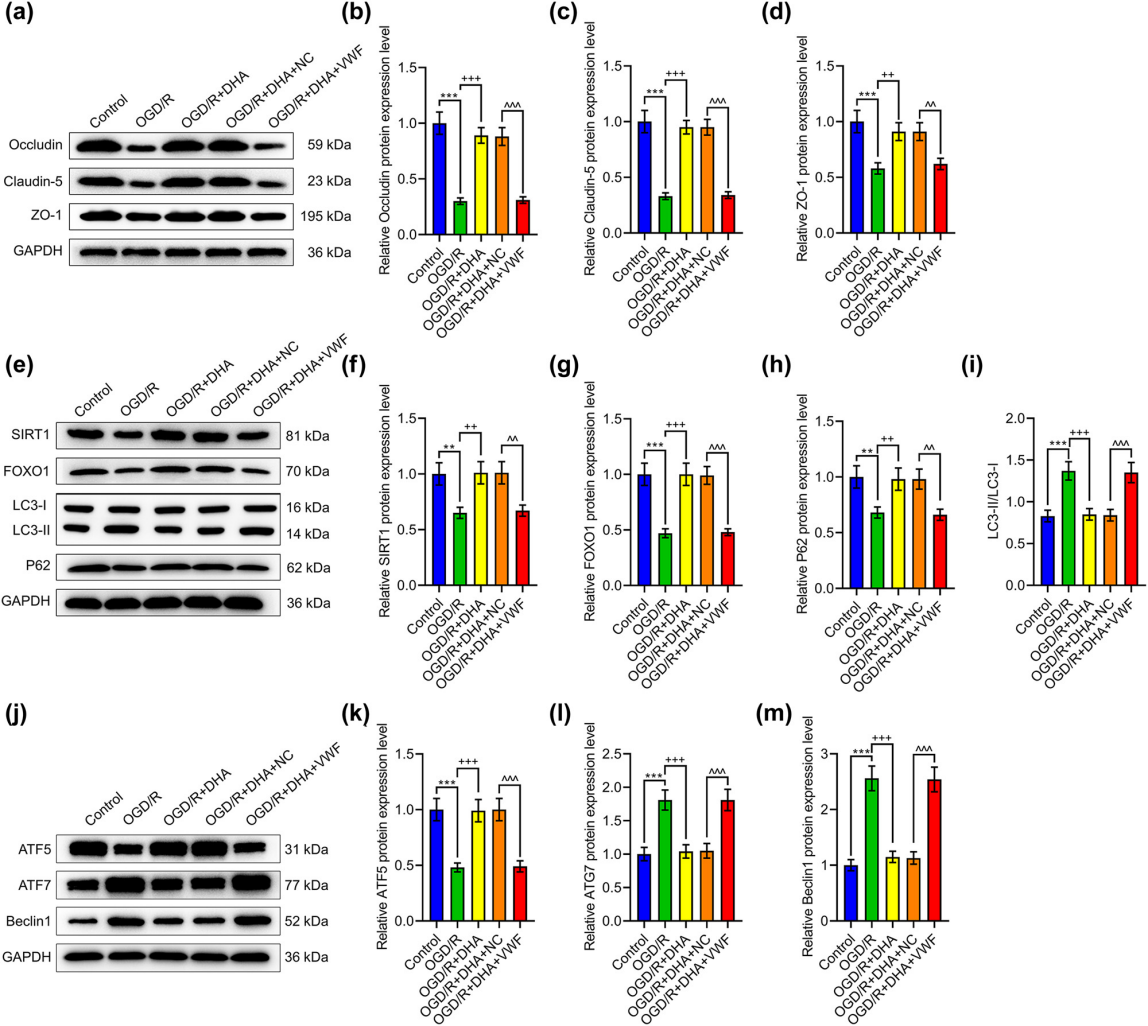
DHA increased tight junction proteins and inhibited autophagy in OGD/R-treated BMVECs via VWF/SIRT1/FOXO1 axis. BMVECs isolated from newborn SD rats were transfected with VWF-overexpressing plasmids or control plasmids. OGD/R exposure was conducted on the cells with or without transfection, followed by 24 h 25 μM DHA treatment. (a–d) Relative protein levels of Occludin, Claudin-5, and ZO-1 in BMVECs were measured by Western blot. GAPDH was used as the loading control. (e–i) Relative protein levels of SIRT1, FOXO1, LC3I, LC3II, and P62 in BMVECs were detected via Western blot. GAPDH was used as the loading control. (j–m) Relative protein levels of ATF5, ATG7, and Beclin1 in BMVECs were measured by Western blot. GAPDH was used as the loading control. ^**^ *p* < 0.01, ^***^ *p* < 0.001 vs Control; ^++^ *p* < 0.01, ^+++^ *p* < 0.001 vs OGD/R; ^^^^ *p* < 0.01, ^^^^^ *p* < 0.001 vs OGD/R + DHA + NC. ATF5, transcription factor 5; ATG7, autophagy-related gene 7; BMVECs, brain microvascular endothelial cells; DHA, dihydroartemisinin; VWF, von Willebrand factor; OGD/R, oxygen–glucose deprivation/reoxygenation; ZO-1, zonula occludens 1; LC3, microtubule associated protein 1 light chain 3 alpha; SIRT1, sirtuin1; FOXO1, forkhead box O1; GAPDH, glyceraldehyde-3-phosphate dehydrogenase; NC, negative control.

To explore the mechanism of DHA in tight junction of BMVECs exposed to OGD/R, we quantified protein expressions of Occludin, Claudin-5, and ZO-1 by Western blot. The results proved that prominent decreases of Occludin, Claudin-5, and ZO-1 expressions were observed in OGD/R-treated BMVECs, which was counteracted after DHA treatment (Figure 3a–d, *p* < 0.01). The protein levels of these three genes were down-regulated by VWF overexpression in the OGD/R- and DHA-treated BMVECs, which reversed the effect of DHA (Figure 3a–d, *p* < 0.01). Furthermore, to elucidate the mechanism by which DHA treatment may suppress autophagy via VWF/SIRT1/FOXO1 axis in OGD/R-treated BMVECs, we measured protein expressions of ATF5, ATG7, Beclin1, LC3-I, LC3-II, P62, SIRT1, and FOXO1 through Western blot. The results demonstrated that the protein expressions of ATF5, SIRT1, FOXO1, and P62 were decreased, and those of ATG7 and Beclin1 as well as LC3-II/LC3-I ratio were increased in the cells exposed to OGD/R, which was reversed by DHA treatment (Figure 3e–m, *p* < 0.01). Notably, VWF overexpression offset the effects of DHA treatment, and strikingly reduced ATF5, SIRT1, FOXO1, and P62 expressions, yet increased the protein expressions of ATG7 and Beclin1 as well as the LC3-II/LC3-I ratio in DHA- and OGD/R-treated BMVECs (Figure 3e–m, *p* < 0.01).

## 4 Discussion

It is documented that reperfusion after cerebral ischemia triggers more intense neurological damage, including excitotoxicity, oxidative stress, and inflammatory responses, in which oxidative stress is the most severe and longest-lasting risk factor [28]. An increasing number of natural compounds have been found to have anti-inflammatory and anti-oxidant biological activities with low toxicity and stable efficacy, which have a wide application prospect in the treatment of cerebrovascular diseases [29–31]. Khan et al. verified that DHA markedly reduces myocardial infarct size and attenuates reperfusion injury in a rat model of myocardial I/R [24]. In a study of lung injury, DHA is able to inhibit tissue fibrosis by reducing oxidative stress [22]. Furthermore, Xiong et al. [32] recently identified that DHA attenuates the damage and improves the function of brains in neonatal SD rats with hypoxic-ischemic brain damage by inhibiting oxidative stress. Similarly, by observing the staining results of brain tissue from rats with cerebral I/R, we found that DHA signally decreased infarct rate, neuronal deficits, and apoptosis, implying that DHA may play a protective role in cerebral I/R injury.

Studies have suggested that high expression of VWF is closely associated with the development and progression of ischemic cerebrovascular diseases through thrombus formation and endothelial dysfunction [9,33,34]. Evidence from Martinez de Lizarrondo et al. confirmed that *N*-acetylcysteine can exert a potent thrombolytic effect in stroke by inhibiting the production of VWF [35]. Moreover, Dong et al. revealed that VWF transcription is decreased by DHA, which is beneficial to vascular homeostasis [23]. In this study, we determined high expression of VWF in rat cerebral cortex after MCAO, and DHA treatment dwindled VWF expression, indicating that VWF may be the molecular target of DHA in cerebral I/R injury.

Research has shown that the development and progression of vasogenic cerebral edema is closely dependent on BBB integrity [36]. In the formation of BBB, tight junction formed between BMVECs is an important factor in maintaining the stability of BBB. Occludin and Claudin-5, as major transmembrane proteins, contribute to the tight junction of endothelial cells, and can interact with cytoplasmic adhesion protein ZO-1 to regulate the structural alterations of tight junction and thus maintain the normal function of BBB [37,38]. The present findings indicated that DHA may stabilize BBB in cerebral I/R injury by enhancing the tight junction. During cerebral I/R, the generation of substantial reactive oxygen species in the ischemic region induces the accumulation of endoplasmic reticulum stress and oxidative stress, so as to prompt excessive autophagy and cause cell death. Shao et al. uncovered that apelin-13 inhibits excessive autophagy through upregulation of P62 and downregulation of LC3B, thereby exerting neuroprotective effects in rats with cerebral I/R injury [39]. P62, as an autophagic substrate, can bind to LC3-II on the internal membrane of autophagic vesicles to form a complex and trigger the autophagic program [40]. In this study, we observed that P62 and ATF5 expressions were reduced, while ATG7 and Beclin1 expressions as well as LC3-II/LC3-I ratio were elevated in the cortex of I/R rats and OGD/R-exposed BMVECs, manifesting that reperfusion-induced excessive autophagy may be the pathological mechanism of BBB dysfunction; however, DHA treatment reversed the above effects of OGD/R. In addition, the following cell function assays identified that DHA improved viability and suppressed apoptosis of OGD/R-induced BMVECs. Based on the above findings, it is suggested that DHA may mitigate the damage of BMVECs and maintain the integrity of BBB through inhibiting autophagy. Nevertheless, whether the molecular mechanism of DHA in these processes is related to VWF has not been anatomized in any study.

SIRT1, as a NAD^+^-dependent deacetylase, is generated through FOXO3 deacetylation, and plays an important role in inhibiting apoptosis, resisting oxidative stress, and delaying cellular senescence [41]. The study by Wang et al. found that piceatannol diminishes oxidative stress in cerebral I/R mice by activating the SIRT1/FOXO1 signaling pathway, thereby alleviating brain damage [42]. Moreover, SIRT1/FOXO1 signaling pathway has been identified as a promising target to prevent VWF-mediated arterial thrombosis [43]. Therefore, we presumed that DHA plays a protective role in cerebral I/R injury by regulating VWF/SIRT1/FOXO1 axis. In order to validate our speculation, we transfected VWF-overexpressing plasmid into BMVECs to carried out recue assays. Interestingly, VWF overexpression neutralized the effect of DHA on the viability, apoptosis, and autophagy of BMVECs as well as on the expressions of tight junction proteins. More importantly, we found that SIRT1 and FOXO1 expressions were enhanced in OGD/R-induced BMVECs after DHA treatment, which was counteracted by VWF overexpression. Although the present findings evidenced that significant effects of DHA treatment can be attenuated by VWF overexpression, whether DHA directly interacts with VWF has not been fully testified. According to a recent study, DHA modulates the E26 transformation-specific (ETS) related gene (ERG) binding with the −56 ETS-binding motif on the human VWF promoter, signifying that DHA decreases VWF expression by the transcription factor ERG [23].

However, on one hand, the singularity of subjects in the *in vitro* experiments is a shortcoming of this study, which will be improved in the next phase of the study. On the other hand, the metabolism of DHA occurs by conjugation with the UDP-glucuronosyltransferase system [44], with an average half-life of 1–2 h [45]. As the degradation of DHA *in vivo* is dependent on its half-life period [24], and animals in this study were subjected to 24 h of reperfusion and 14 days of recovery, it might be necessary to increase the administration times during the treatment.

## 5 Conclusion

To conclude, we provide the first evidence that DHA may reduce BBB damage by inhibiting VWF and activating SIRT1/FOXO1 signaling pathway, thereby ameliorating cerebral I/R injury in rats.
